# Resynthesized lines from domesticated and wild *Brassica* taxa and their hybrids with *B. napus* L.: genetic diversity and hybrid yield

**DOI:** 10.1007/s00122-012-2036-y

**Published:** 2013-01-18

**Authors:** Tobias Jesske, Birgit Olberg, Antje Schierholt, Heiko C. Becker

**Affiliations:** 1 Department of Crop Sciences, Georg August Universität Göttingen, Von Siebold Strasse 8, 37075 Göttingen, Germany; 2 Present Address: Lantmännen SW Seed Hadmersleben GmbH, Kroppenstedter Str. 4, 39387 Oschersleben, Germany

## Abstract

Resynthesized (Resyn) *Brassica napus* L. can be used to broaden the genetic diversity and to develop a heterotic genepool for rapeseed hybrid breeding. Domesticated vegetable types are usually employed as *B*. *oleracea* parents. We sought to evaluate the potential of wild species as parents for Resyn lines. Fifteen Resyn lines were derived by crossing wild *B*. *oleracea* ssp. *oleracea* and oilseed *B. rapa*, and 29 Resyn lines were generated from 10 wild *Brassica* species (*B. bourgaei, B. cretica, B. incana, B. insularis*, *B. hilarionis*, *B. macrocarpa*, *B. montana*, *B. rupestris*, *B. taurica*, *B. villosa*). Genetic distances were analyzed with AFLP markers for 71 Resyn lines from wild and domesticated *B. oleracea*, and compared with 55 winter, spring, vegetable, and Asian *B. napus* genotypes. The genetic distances clearly showed that Resyn lines with wild species provide a genetic diversity absent from the breeding material or Resyn lines from domesticated species. Forty-two Resyn lines were crossed with one or two winter oilseed rape testers, resulting in 64 hybrids that were grown in one year and four locations in Germany and France. The correlation between hybrid yield and genetic distance was slightly negative (*r* = −0.29). Most of the hybrids with Resyn lines from wild *B. oleracea* were lower in yield than hybrids with Resyn lines from domesticated *B. oleracea*. It is promising that Resyn lines descending from unselected wild *B. oleracea* accessions produced high-yielding hybrids when crossed with adapted genotypes: these Resyn lines would be suited to develop heterotic pools in hybrid breeding.

**Electronic supplementary material:**

The online version of this article (doi:10.1007/s00122-012-2036-y) contains supplementary material, which is available to authorized users.

## Introduction

*Brassica napus* L. is an allopolyploid (amphidiploid) species that resulted from interspecific hybridization between *B. oleracea* (C genome) and *B. rapa* (A genome). Allopolyploidy is an evolutionary process whereby two or more genomes are combined by spontaneous inter-specific or inter-generic hybridization, followed by chromosome doubling. Several important crops, such as bread and durum wheat, oat, cotton, and coffee, are allopolyploid (Feldman and Levy 2005).

In *B. napus* it is possible to produce ‘resynthesized’ (Resyn) genotypes via an artificial cross between the parental species *B. oleracea* and *B. rapa*. Resyn rapeseed genotypes have been used for many years to broaden the genetic variation of oilseed rape: an overview of this strategy of introgression of single traits is given by Qiong et al. (2009). Becker et al. (1995) suggested the use of Resyn lines to establish a genetically diverse winter oilseed rape gene pool that can be used in hybrid breeding. Because only a few *B. rapa* and *B. oleracea* genotypes led to the first spontaneous *B. napus* in medieval times (Iñiguez-Luy and Federico 2011), the use of a broad range of *B. rapa* and *B. oleracea* taxa would increase the diversity in Resyn lines, and consequently, in the *B. napus* gene pool. Diversity in the *B. napus* gene pool is one requirement for successful hybrid breeding programs, based on the assumption of a positive correlation between heterosis and genetic distance (Falconer and Mackay 1996). Different approaches have been used to broaden genetic variation in the *B. napus* gene pool with genetically distant material, such as Kebede et al. (2010), who described the introgression of winter rapeseed cultivars into the Canadian spring rapeseed gene pool. Zou et al. (2010) suggested exploiting intersubgenomic heterosis in *B. napus* through the partial introgression of subgenomic components from different *Brassica* species and Qian et al. (2009) analyzed Chinese semi-winter lines as distant parental lines in European winter oilseed rape hybrid programs.

The use of Resyn lines as hybrid parents and the resulting increase in hybrid yield due to heterosis was previously described for spring *B. napus* (Girke et al. 2001; Udall et al. 2004; Seyis et al. 2006) and winter oilseed rape (Girke et al. 2011b). Nearly all of the Resyn lines in these studies originated from interspecific crosses of domesticated *B. rapa* and *B. oleracea* genotypes. However, the domesticated *B. oleracea* vegetable types had been selected for vegetable yield and quality, not for seed yield. The *Brassica oleracea* group contains not only cultivated and wild *B. oleracea*, but also nine species that show morphologically a wide range of diversity (*B. bourgaei, B. cretica, B. incana, B. insularis*, *B. hilarionis*, *B. macrocarpa*, *B. montana*, *B. rupestris*, and *B. villosa*). All of them have the same chromosome number (*n* = 9), can be crossed with each other, and produce fertile hybrids (Hanelt 2001).

In the present study, Resyn lines were obtained from crosses of *B. rapa* varieties with wild *B. oleracea* taxa, and the resulting Resyn lines were used as hybrid parents. While domesticated vegetables were selected for qualities such as taste or shape, seed number is a major fitness component for wild species. Therefore, we assumed that Resyn lines with a wild species serving as the donor of the C genome would have a relatively high seed performance compared with Resyn lines from *B. oleracea* vegetable types. The objectives of this study were to (1) analyze the genetic distances in collections of winter, spring, vegetable, and Asian *B. napus* genotypes (*n* = 55) in comparison to 71 Resyn lines, of which 44 originated from crosses with wild *B. oleracea* taxa and (2) evaluate the yield of hybrids derived from crosses of 42 Resyn lines with one or two male sterile *B. napus* tester lines.

## Materials and methods

### Germplasm

The collection of 126 genotypes consisted of 55 *B. napus* varieties (Table 1; ESM 1), and 71 Resyn lines (ESM 2). The diverse set of *B. napus* varieties included winter, spring, Asian, and vegetable varieties. These genetic groups were composed of modern varieties such as the spring-type ‘Favorite’ and ‘Siesta’ varieties, with 00 quality (zero erucic acid and low glucosinolate content in the seeds), and obsolete varieties including ‘Mansholts Hamburger’ and ‘Zachodni’. Chinese, Turkish, and vegetable *B. napus* varieties completed the broad genetic material.

**Table 1:** *Brassica napus* varieties and lines

Genotype	Type/origin	Seed quality	Year of release	Genotype	Type/origin	Seed quality	Year of release
Aphid resistant rape	W, V			Mazowieki	S	++	~1945
Alesi	W	00	2004	Mlochowski	S	++	~1945
Billy	W	00	2005	Nugget	S	++	1961
Campari	W, F	00	1996	Petranova	S	++	1963
Digger	W	00	2002	Regina	S	++	1942
Emerald	W, F	00	1973	Siesta	S	00	2003
Express 617	W	00	1993	Svaloefs gulle	S	++	1969
Favorite	W	00	2006	Tanka	S	++	1963
Gießener Höhenraps	W	++	<1945	Tira	S	++	1972
Jet Neuf	W	0+	1977	Topas	S	00	1981
Ladoga	W	00	2005	Westar	S	00	1982
Lembkes Normal	W	++	1941	Zachodni	S	++	~1945
Mansholt 54^a^	W	++	<1945	Eskisehir	S, T		
Mansholts Hamburger	W	++	1899	Turhal	S, T		
Mosa	W, F	00	2001	Yenisehir	S, T		
Nikos	W, F	00	2000	Ganyu 3	A	++	1977
Norde	W	++	1968	Italy	A	++	
Oase	W	00	2004	Linyou 5	A	++	
Samourai 11.4^a^	W	00		Zhenyou 11	A	++	
Samourai	W	00	1989	Xiangyou 11	A	00	
Sollux	W	++	1973	87-50182	A	++	
Viking	W	00	2002	Brauner Schnittkohl	V (leaf)		< 1945
Tester A	W, mS	00	1999	Goldgelber zarter butter	V (leaf)		< 1945
Tester B	W, mS	00	2009	Grüner Schnittkohl	V (leaf)		< 1945
Bronowski	S	+0	~1945	Mecklenburger Weiße	V (sw)		<1945
Golden	S	++	1954	MB6-BRS-039	V (leaf)		
Heros	S	00	2000	Wilhelmsburger Steckrübe	V (sw)		
Licosmos	S	00	1996				

High (+) or low (0) content of erucic acid and glucosinolates in the seeds

*W* winter, *S* spring, *T* Turkish, *A* Asian, *mS* male sterile line, *F* fodder type, *V* vegetable, *V*(*sw*) vegetable (swede), *V*(*leaf*) vegetable (leafy cabbage)

^**a**^Doubled haploid line of the respective variety

The Resyn lines were subdivided into domesticated Resyn lines (R_DOM_ *n* = 27) and wild Resyn lines (R_WILD_ *n* = 44), according to the origin of their *B. oleracea* parental genotypes (ESM 2). The R_DOM_ lines originated from crosses of diverse domesticated *B. oleracea* taxa (vegetable forms) that were described earlier by Girke et al. (2011a), with the exception of eight lines that were obtained from the Justus-Liebig-Universität Gießen, Germany, and line ‘RS 8/6’, from Freie Universität Berlin, Germany (ESM 2).

The R_WILD_ lines were derived from interspecific crosses of *B. rapa* oilseed varieties (A genome) with 11 wild *B. oleracea* taxa (C genome). Depending on the C genome donator, the group of R_WILD_ lines was further subdivided into R_WTYPE_ and R_WSPEC_ Resyn lines. Twenty-nine R_WSPEC_ lines originated from crosses of *B. rapa* with ten wild species of the *B. oleracea* or C genome group. The names of the R_WSPEC_ lines were composed of their parental genotypes, where the first two letters describe the paternal species: *B. bourgaei* (BO)*, B. cretica* (CR)*, B. incana* (IN)*, B. insularis* (IS), *B. hilarionis* (HI), *B. macrocarpa* (MA), *B. montana* (MO), *B. rupestris* (RU), *B. taurica* (TA), and *B. villosa* (VI). The last letter stands for the *B. rapa* genotype: ‘Y’ for ‘Yellow Sarson’ (spring oilseed) or ‘L’ for ‘Largo’ (winter oilseed). Fifteen R_WTYPE_ lines were obtained from crosses with *B. oleracea* ssp. *oleracea* (wild *B. oleracea*) with winter *B. rapa* oilseed variety ‘Largo’ and breeding line ‘NPZ 00’. The names of the R_WTYPE_ lines are composed of the letter ‘J’ and a number (ESM 2). Taxonomic classification was carried out according to Hanelt (2001).

### Genomic DNA extraction and amplified fragment length polymorphism (AFLP) analysis

Leaf material was harvested in the greenhouse from one plant per genotype. DNA was extracted using the Nucleon Phytopure plant extraction Kit (GE Healthcare, Illustra™). AFLP procedures (Vos et al. 1995) were modified for multiplex PCR in accordance with Ecke et al. (2010). Twenty primer combinations were tested and three highly polymorphic combinations were selected (M50/E37, M50/E40, and M50/E42), which revealed 471 polymorphic AFLP fragments. The detection of AFLP fragments was performed on the ABI PRISM 3130 Genetic Analyzer (Applied Biosystems) with a 36-cm capillary array, and the GeneScan-500 LIZ (Applied Biosystems) was set as standard. The data were semi-automatically scored by GeneMapper v4.0 (Applied Biosystems) for the presence or absence of the relevant bands.

Genetic distances among genotypes based on AFLP markers were estimated in accordance with Jaccard (1908), as suggested by Link et al. (1995) for dominant markers. The cluster analysis was performed using the unweighted pair group method with arithmetic mean. Dendrograms were verified with cophenetic correlations as a measure of goodness of fit (Sneath and Sokal 1973), which revealed a correlation of *r* = 0.92. The clusters were validated via bootstrap analysis (Felsenstein 1985). The principal coordinate analysis was performed according to Backhaus et al. (1990). All genetic distance analyses were carried out with FreeTree (Hampl et al. 2001) and NTSYSpc 2.1 (Rohlf 2000), and dendrograms were edited with TreeView software (Page 1996).

### Hybrid seed production

Resyn lines were used as pollen source for test cross seed production with maternal tester lines ‘MSL 007’ and ‘RNX 4621’, named ‘Tester A’ and ‘Tester B’. The tester lines and the fertile forms of the tester lines (‘fertile line tester A’ and ‘fertile line tester B’) were received from Norddeutsche Pflanzenzucht Hans-Georg Lembke KG and Syngenta Seeds, respectively. In the 2008–2009 season, hybrids with ‘Tester B’ were produced in the field in Biemsen, Germany, with ‘Tester A’ in Malchow, Germany, and with both testers at the Georg-August-University Göttingen in the greenhouse. In the field, hybrid seeds were produced as described by Girke et al. (2011b), with minor modifications. Since it could not be expected that all Resyn lines would be winter hardy, all Resyn lines at locations Malchow and Biemsen were sown in the greenhouse and were vernalized in a climate chamber, whereas the maternal tester lines were sown directly in the field in the autumn of 2008. The vernalized Resyn pollinator plantlets were transferred to the field in the spring of 2009 and were planted in the rows between the tester lines. Before flowering, each plot was covered by an insect-proof net and pollinator insects were added (Girke et al. 2011b). In addition to the hybrid production in the field, all test cross combinations were produced in the greenhouse (Göttingen), where three plants each from the Resyn pollinator and maternal lines were isolated with bags during flowering. However, hybrid seed production in the field was hampered, mainly by poor development of most Resyn lines: hybrid seeds could not be produced from all combinations, and only a limited number of hybrid seeds was obtained.

### Field trials

Sixty-four test hybrids and three check varieties were grown in a randomized block design without replication at four locations during the 2009–2010 growing season. Plot sizes differed from 11.25 to 18.0 m². The trials were performed at Hohenlieth (Northern Germany, near the Baltic sea), Einbeck and Göttingen (Central Germany), and Resson sur Metz (Central France). Due to seed shortage, a reduced set of 39 hybrids was tested at five additional locations (Thüle and Rosenthal in Central Germany, Biemsen in Northern Germany, Hohenlieth 2, and Thriplow in the United Kingdom). The results of these trials appear in ESM 3, while the data from the larger hybrid test set are presented throughout this publication. The following check varieties were included: hybrid variety ‘Visby’, ‘fertile line tester A’, and ‘fertile line tester B’.

The following parameters were recorded in the field trials: beginning of flowering (number of days in 2010), development before and after winter (score 1–9, score 1 = loss of most plants to 9 = plot is complete), plant height at the end of flowering (in cm), and lodging (score 1–9, no lodging to heavy lodging). Winter hardiness was estimated as the difference between the scores of before and after winter development, with smaller numbers indicating better winter hardiness. Seed yield was determined in dt ha^−1^ (relative to 91 % dry matter). As quality parameters of the harvested seeds, the oil (%, relative to 91 % dry matter), protein (%, relative to 91 % dry matter), and glucosinolate (μmol g^−1^, relative to 91 % dry matter) content were measured using near-infrared reflectance spectroscopy (NIRS; Reinhard 1992) via the NIRS 6500 (Foss GmbH, Slangerupgade 69, DK-3400 Hillerød), using the calibration raps2010.eqa provided by VDLUFA Qualitätssicherung NIRS GmbH (Am Versuchsfeld 13, D-34128 Kassel, Germany). The thousand-seed weight (g) was determined.

### Statistical analyses of the field trials

The statistical analysis of the field trials, estimations of correlations, least significant differences, heritability (*H*²), and tests of significance were performed using PLABSTAT software (Utz 2007). Analysis of variance (ANOVA) of the field trials was calculated with the following model: *Y*_ij_ = μ + *g*_i_ + *l*_j_ + (*gl*)_ij_, where *Y*_ij_ is defined as the observation of genotype i at location j, μ is the overall mean, *g*_i_ is the effect of genotype i (for i = 1, …, 67), *l*_j_ is the effect of location j (for j = 1, …, 4), and (*gl*)_ij_ is the corresponding interaction effect, including experimental error. The ANOVA was calculated as a mixed model, where the effect of the location was considered random and the effect of the genotype was considered fixed.

## Results

### Genetic distance analyses

The mean genetic distance of all 15,750 paired comparisons was 0.54, with a range of minimum 0.09 and maximum 0.81 in all pairwise comparisons (Table 2). The genetic distances between the *B. napus* genetic groups (winter, spring, Asian, and vegetable), and R_DOM_ and R_WILD_ lines, were higher than the within-group distances. The highest mean between-group genetic distances involved Resyn lines (0.61–0.62), whereas the smallest genetic distance was observed between winter and vegetable *B. napus* (0.34). All genetic group comparisons with R_DOM_ lines revealed higher mean genetic distances (0.45–0.53) than those with *B. napus* varieties (winter, spring, Asian, vegetable). The mean within-group distance in *B. napus* varieties was lower than that in Resyn lines: 0.29 in winter, 0.28 in spring, 0.37 in Asian, and 0.41 in vegetable *B. napus*.

**Table 2:** Mean and range of the genetic distances within and between genotype groups

Genotype groups	*N* ^a^	Mean	Minimum	Maximum
W × W	506	0.29	0.12	0.42
W × S	437	0.37	0.22	0.51
W × A	138	0.39	0.26	0.52
W × V	161	0.34	0.19	0.54
W × R_DOM_	621	0.50	0.26	0.71
W × R_WILD_	1012	0.61	0.44	0.79
W × R_ALL_	1633	0.57	0.26	0.79
S × S	342	0.28	0.19	0.44
S × A	114	0.46	0.33	0.54
S × V	133	0.38	0.26	0.50
S × R_DOM_	513	0.53	0.33	0.71
S × R_WILD_	836	0.61	0.44	0.76
S × R_ALL_	1349	0.58	0.33	0.75
A × A	30	0.37	0.29	0.43
A × V	42	0.41	0.39	0.52
A × R_DOM_	162	0.45	0.35	0.69
A × R_WILD_	264	0.62	0.49	0.79
A × R_ALL_	426	0.59	0.35	0.79
V × V	42	0.41	0.23	0.50
V × R_DOM_	189	0.51	0.28	0.70
V × R_WILD_	308	0.61	0.44	0.78
V × R_ALL_	497	0.57	0.28	0.78
*B. napus* × *B. napus*	2970	0.35	0.12	0.54
*B. napus* × R_DOM_	1485	0.52	0.26	0.71
*B. napus* × R_WILD_	2420	0.61	0.44	0.79
*B. napus* × R_ALL_	3905	0.58	0.26	0.79
R_DOM_ × R_DOM_	702	0.55	0.18	0.75
R_WILD_ × R_WILD_	1892	0.59	0.09	0.79
R_DOM_ × R_WILD_	1188	0.61	0.35	0.81
R_ALL_ × R_ALL_	4970	0.59	0.09	0.81

*W* winter, *S* spring, *A* Asian, *V* vegetable, R_DOM_ Resyn line derived of domesticated *B. oleracea*, R_WILD_ Resyn line derived of *B. oleracea* wild-types and wild species, R_ALL_ all Resyn lines, *B.n. cultivars B. napus* = combination of W, S, A, and V

^a^Number of paired comparisons

The winter oilseed rape varieties ‘Billy’ and ‘Oase’ had the smallest genetic distance (0.12), while varieties ‘Campari’ and ‘Mansholt 54’ had the highest genetic distance (0.42). The highest variation in spring *B. napus* occurred between ‘Bronowski’ and ‘Zachodni’ (0.44), whereas ‘Golden’ and ‘Licosmos’ were associated with a genetic distance of only 0.19. In the group of Asian *B. napus*, the ‘Italy’ and ‘Xiangyou 11’ varieties revealed the smallest (0.29) genetic distance, while ‘Linyou 5’ and ‘Ganyu 3’ had the highest genetic distance (0.43). The variation in the *B. napus* vegetable forms ranged from 0.23 (between ‘Brauner Schnittkohl’ and ‘Goldgelber Zarter Butter’) to 0.50 (between ‘Grüner Schnittkohl’ and ‘Wilhelmsburger Steckrübe’). The highest within-group variation occurred in the R_WILD_ lines. The R_WILD_ lines ‘MAY 1’ and ‘MOY 1’ had the highest genetic distance (0.79), while lines ‘J166’ and ‘J400’ had the smallest genetic distance (0.09) within this group. All pairwise comparisons are available by request.

**Fig. 1:**
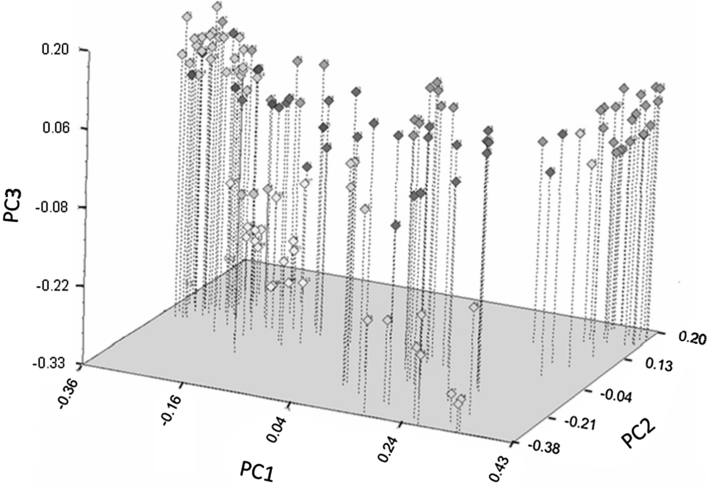
Principal coordinate analysis of 55 *B. napus* cultivars and 71 Resyn lines. The first three principal coordinates (PC) explained 13.36, 4.70, and 3.76 % of the variation. Symbol colors of cultivar genotype groups are winter (*light blue*), vegetable (*blue*), spring (*yellow*), Asian (*orange*), Turkish (*pink*). The groups of Resyn lines are colored as follows: R_DOM_ (*violet*), R_WTYPE_ (*light green*), R_WSPEC_ (*green*) (color figure online)

In the principal coordinate analysis of 55 cultivated forms and 71 Resyn lines, the first three principal coordinates explained 21.8 % of the variation (Fig. 1). The three-dimensional diagram allows the discrimination of genetic groups with the varieties (winter, spring, Asian, vegetable) on the upper left of the principal coordinate analysis diagram clearly distinguishable from the R_WILD_ lines. Overall, the group of Resyn lines showed the largest genetic distances, and various subgroups occurred within the R_WILD_ genotypes that can be traced back to the corresponding parental genotypes: R_WTYPE_, R_WSPEC_, ‘Yellow Sarson’, and ‘Largo’. Two subgroups were differentiated within the R_WSPEC_ lines (Fig. 1, green): one group originated from crosses of *Brassica* wild species with ‘Largo’, a winter *B. rapa* variety (upper center), and the other group consisted of crosses of *Brassica* wild species with the spring *B. rapa* ‘Yellow Sarson’ (upper right corner). Both groups were distinguished on the basis of the first principal coordinate. Corresponding subgroups were detected within the R_WTYPE_ Resyn lines (Fig. 1, light green). Two R_WTYPE_ Resyn lines, ‘OLY 1’ and ‘OLY 2’, were grouped with ‘Yellow Sarson × R_WSPEC_’. Winter and spring *B. napus* were clearly distinguishable. Three Turkish varieties grouped with the spring *B. napus*. The Asian and vegetable *B. napus* genotypes did not form separate groups and were placed near the winter *B. napus*.

**Fig. 2:**
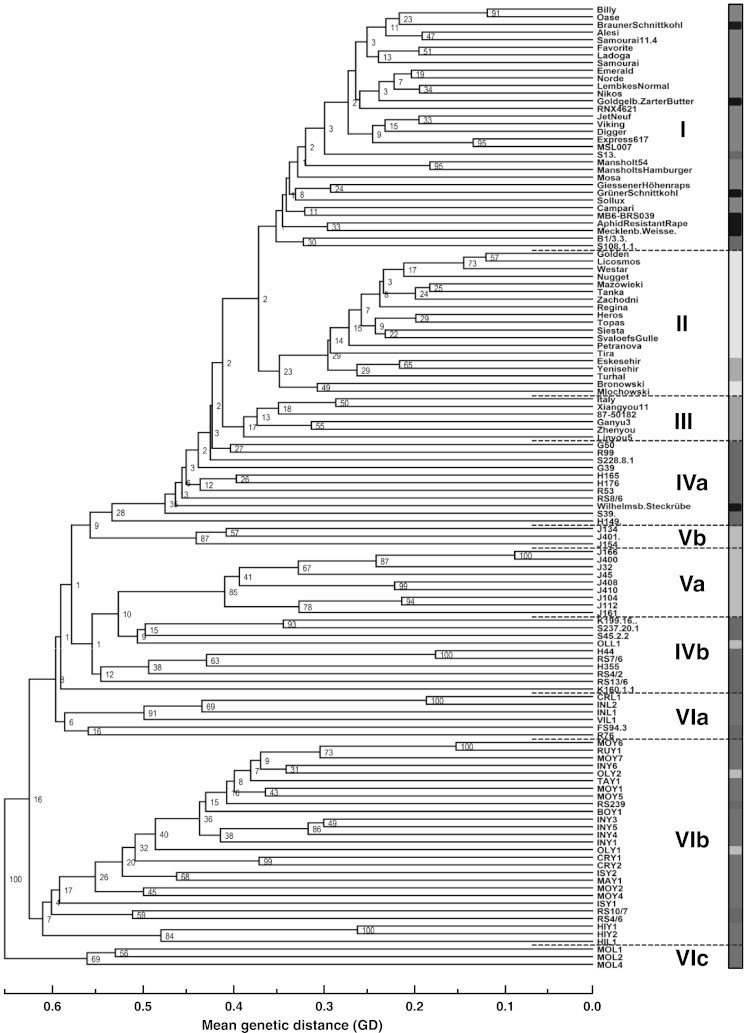
Dendrogram of 55 *B. napus* cultivars and 71 Resyn lines. Colors of cultivar genotype groups are winter (*light blue*), vegetable (*blue*), spring (*yellow*), Asian (*orange*), Turkish (*pink*); and colors of the groups of Resyn lines are R_DOM_ (*violet*), R_WTYPE_ (*light green*), R_WSPEC_ (*green*). The roman numerals indicate groups of genotypes (color figure online)

The pattern of genetic distances within and between genotype groups is shown in more detail in the dendrogram from the cluster analysis (Fig. 2). Here, three clusters (I, II, III) formed a subgroup that included nearly all cultivated *B. napus* genotypes of the winter, spring, Asian, and vegetable types. Cluster I included all winter *B. napus*, three R_DOM_ lines (‘S 13’, ‘B1/3.3’, and ‘S 108.1.1’), and all vegetables, with the exception of the ‘Wilhelmsburger Steckrübe’, which was located in cluster IVa. Three Turkish varieties formed cluster II with the spring *B. napus* (Figs. 1, 2), a pattern that can be explained by their origin in the European spring *B. napus* breeding programs (M. Kemal Gül 2012, personal communication). The Asian genotypes formed a separate cluster (cluster III).

Clusters I and II, containing the winter and spring genotypes, respectively, were separated at a mean genetic distance of 0.37, while clusters II and III (Asian group) had a genetic distance of 0.41, respectively. Clusters VIa, VIb, and VIc showed the highest distances to the cultivated genotypes in clusters I, II, and III. These clusters included all R_WSPEC_ lines, with the C genome of wild *Brassica* species. Clusters VIa, VIb, and VIc separated at mean genetic distances of 0.60, 0.63, and 0.65, respectively. Cluster VIa included R_WSPEC_ genotypes and two R_DOM_ lines (‘FS 94.3’ and ‘R 76’), whereas cluster VIb integrated three R_DOM_ lines (‘RS 4/6’, RS 10/7’, and ‘RS 239’) and two R_WTYPE_ lines (‘OLY 1’ and ‘OLY 2’), which were obtained from crosses with *B. oleracea* ssp. *oleracea*. Most R_WTYPE_ lines were located in clusters Va and Vb. Cluster Va was separated from the *B. napus* varieties at a genetic distance of 0.55, while cluster Vb formed a subgroup with the R_DOM_ lines in cluster IVa, separated at a genetic distance of 0.58. The majority of the R_DOM_ lines grouped in clusters IVa and IVb, which were located between the subgroups of the varieties (clusters I, II, and III) and the R_WSPEC_ lines (cluster VI), which were obtained from crosses with wild *Brassica* species. R_DOM_ line ‘H 149’ was positioned adjacent to cluster IVa, while the rest of the R_DOM_ lines were assigned to clusters I (winter *B. napus*) and VI (R_WSPEC_).

### Hybrid yield

Genotype and location were significant sources of variation (*P* = 0.01) for the test hybrids for all traits except winter hardiness (Table 3). The heritabilities of yield, oil content, protein content, erucic acid content, and glucosinolate content were high (>0.75). Winter hardiness and lodging had low heritability (*H*² = 0.16 and 0.41, respectively).

**Table 3:** Mean squares and tests of significance from the ANOVA and heritabilities (*H*²)

Source of variation	Genotype (G)	Location (L)	G × L	*H*²
Seed yield (dt ha^−1^)	14.89**	58.25**	20.19	0.75
Thousand-seed weight (g)	0.08**	0.15**	0.04	0.86
Plant height (cm)	25.92**	95.84**	59.89	0.63
Beginning of flowering (days)	7.31**	118.52**	4.84	0.82
Winter hardiness	0.06	0.35**	1.09	0.16
Lodging	0.22**	0.48**	0.98	0.41
Seed oil (%)	1.26**	2.23**	0.71	0.88
Erucic acid (%)	36.82**	33.54**	12.55	0.92
Protein (%)	0.46**	0.60**	0.53	0.78
Glucosinolate (μmol g^−1^)	87.62**	1.37**	21.84	0.94

Genotypes (G) were hybrids of Resyn lines with ‘Tester A’ and ‘Tester B’, grown at four locations (L) in the season 2009–2010

Probability levels are indicated as follows: ** for *P* = 0.01; * for *P* = 0.05 and ^+^ for *P* = 0.1

**Fig. 3:**
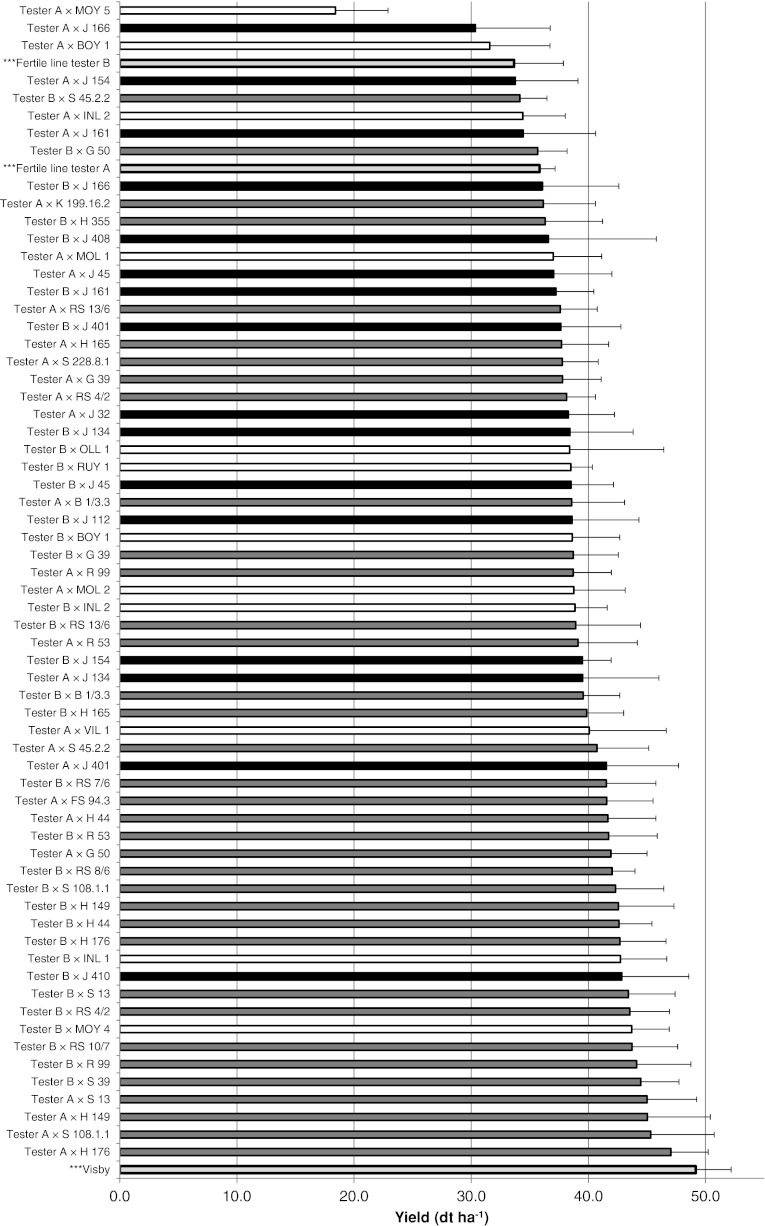
Mean seed yield (dt ha^−1^) of hybrids and checks evaluated at four locations in the season 2009–2010. Colors indicate the genetic group of the Resyn line: R_DOM_ (*grey*), R_WTYPE_ (*black*), R_WSPEC_ (*white*). The checks are colored in *light gray* and marked with asterisks. Standard errors are included as *horizontal lines*

The yields of check varieties ‘fertile line tester A’ (35.8 dt ha^−1^) and ‘fertile line tester B’ (33.7 dt ha^−1^) were lower than the mean yield of the test hybrids (39.2 dt ha^−1^), while the yield of check variety ‘Visby’ exceeded all test hybrids (49.2 dt ha^−1^; Fig. 3; Table 4; ESM 3). Only three hybrids, ‘Tester A × MOY 5’, ‘Tester A × J 166’, and ‘Tester A × BOY 1’, exhibited a yield that was lower than ‘fertile line tester B’.

**Table 4:** Mean yield and agronomic parameters of hybrids and checks, evaluated at four locations in the season 2009–2010

Genetic groups	Yield (dt ha^−1^)	Oil (%)	Erucic acid (%)	Protein (%)	GSL	TSW (g)	Plant height (cm)	BF	Winter hardiness^a^	Lodging (1–9)
Checks										
‘Fertile line tester A’	35.8	45.4	1.3	17.9	16.9	4.4	142.5	117.3	1.8	1.3
‘Fertile line tester B’	33.7	42.1	2.3	18.5	21.6	4.7	150.0	121.4	2.3	3.0
‘Visby’	49.2	43.6	0.0	17.0	15.0	4.8	157.5	117.0	2.0	1.7
Mean of checks	39.6	43.7	1.2	17.8	17.8	4.6	150.0	118.6	2.0	2.0
Hybrids										
Mean of hybrids with R_DOM_	40.8^**b**^	43.7^**b**^	13.8^**b**^	18.7^**b**^	38.1^**b**^	4.5^**b**^	157.3^**c**^	114.6^**bc**^	2.4^**b**^	2.4^**b**^
Mean of hybrids with R_WSPEC_	36.5^**c**^	41.8^**c**^	11.5^**b**^	19.6^**c**^	43.5^**b**^	4.8^**c**^	159.3^**c**^	116.3^**c**^	2.8^**c**^	2.5^**b**^
Mean of hybrids with R_WTYPE_	37.5^**c**^	42.7^**d**^	14.3^**b**^	18.6^**b**^	37.4^**b**^	4.6^**c**^	149.5^**b**^	113.1^**b**^	3.0^**c**^	3.3^**c**^
Mean of hybrids	39.2	43.1	13.5	18.8	38.9	4.6	155.7	114.6	2.6	2.7
Overall										
Least significant difference (LSD *P* = 0.05)	6.3	1.2	4.9	1.0	6.5	0.3	10.8	3.6	1.5	1.6
Minimum	18.4	40.4	0.0	17.0	15.0	4.0	137.5	106.7	1.8	1.3
Maximum	49.2	46.2	25.1	21.0	60.2	5.4	168.8	121.4	4.5	5.0

*GSL* glucosinolate content (μmol g^−1^); * TSW* thousand-seed weight; * BF* beginning of flowering in number of days in the year 2010; R_DOM_ Resyn lines derived of domesticated *B. oleracea*; R_WSPEC_, Resyn lines derived of *B. oleracea* wild species; R_WTYPE_, Resyn lines derived of *B. oleracea* wild-types

^a^For the definition of winter hardiness see “Materials and methods”

^b–d^different letters indicate significant differences between genetic groups (*P* = 0.05, LSD Test)

The mean seed yield of hybrids originating from R_DOM_ lines was significantly higher (40.8 dt ha^−1^; *P* = 0.05; Table 4) than that of hybrids derived from R_WTYPE_ (37.5 dt ha^−1^) or R_WSPEC_ (36.5 dt ha^−1^) lines (Table 4). Five hybrids derived from R_WILD_ lines had a yield that was higher than 40 dt ha^−1^ (‘Tester A × VIL 1’, ‘Tester A × J 401’, ‘Tester B × INL 1’, ‘Tester B × J 410’, and ‘Tester B × MOY 4’). The yield of hybrid ‘Tester A × H 176’ was nearly as high as that of the check variety ‘Visby’: ‘H 176’ is an R_DOM_ genotype (Fig. 3). Twenty-two Resyn lines were used as parental lines with both tester lines ‘A’ and ‘B’, and a significant correlation (*r* = 0.45) was estimated for yield from the resulting hybrids.

### Seed quality

The seed oil content of most test hybrids with R_WTYPE_ and R_WSPEC_ lines ranged between 40 and 43 % (Table 4; ESM 3); most test crosses with a higher seed oil content resulted from crosses with R_DOM_ lines. ‘Fertile line tester B’ had a low oil content (42.1 %) compared with ‘fertile line tester A’ (45.4 %) and ‘Visby’ (43.6 %). Three test crosses of ‘Tester A’ and R_DOM_ lines ‘H 149’, ‘H 176’, and ‘S 13’, with oil contents of 45.5, 45.6, and 46.2 %, respectively, exceeded the ‘high-oil’ line ‘fertile line tester A’. The mean protein content of the test crosses with Resyn lines was higher than the mean of check varieties, with the highest protein content in ‘Tester A × MOL 2’ (21.0 %). Erucic acid and glucosinolate contents varied significantly in the test hybrids (Table 3). Both tester lines had low glucosinolate and erucic acid contents, as did some of the test hybrids with R_DOM_ lines, such as ‘FS 93.4’, and ‘G 50’, with low erucic acid content, or ‘S 39’, with low glucosinolate content (Table 4; ESM 3).

### Winter hardiness and lodging

For winter hardiness, the descriptive value expressed the difference in the parameter scores before and after winter. The low scores of most hybrids therefore reflect relatively good winter hardiness, with a mean difference of 2.6 for checks and of hybrids (Table 3; ESM 3). The hybrids ‘Tester B × J 161’, ‘Tester A × S 45.2.2’, and ‘Tester B × J 408’ exhibited the least winter hardiness. Most hybrids did not lodge to a greater extent, with the majority ranking within the range of the check varieties. Exceptions were the hybrids of Resyn line ‘J 166’ with both testers, and ‘Tester B × J 401’, all of which had a mean score of five. These three hybrids did not stand out for plant height (ESM 3), but ranked around the mean value of all Resyn hybrids (155.7 cm; Table 4).

## Discussion

### Genetic divergence

*B. napus* is a comparatively young species with a narrow genetic base. It is of polycentric origin: the original hybridization events between *B. rapa* and *B. oleracea* occurred on more than one occasion, and there is evidence that the C genome was contributed from various *Brassica* species. Nevertheless, it is very unlikely that this spontaneous hybridization happened very often (Song and Osborn 1992; Allender and King 2010; Iñiguez-Luy and Federico 2011). The *B. napus* gene pool was further narrowed by selection for quality traits, because the same two donors had been used worldwide to obtain zero erucic acid and low glucosinolate content (Becker et al. 1999; Seyis et al. 2003; Hasan et al. 2006; Bus et al. 2011).

The low overall mean genetic distance in varieties (0.35) and in the winter, spring, Asian, and vegetable subgroups (0.29, 0.28, 0.37, and 0.41, respectively) corroborated the results of earlier studies. Becker et al. (1995) estimated in-group genetic distances of 0.20 and 0.21 in winter and spring *B. napus*, respectively, while Girke et al. (2011a) reported genetic distances of 0.21, 0.23, and 0.28 for winter, spring, and Asian variety groups, respectively.

The genetic diversity in the groups of varieties was lower than that in the Resyn lines (Table 2). The mean genetic distances in the Resyn lines (0.59), as well as the distance between the R_DOM_ and R_WILD_ subgroups, was higher than in the genotype groups of varieties, indicating a reduction in genetic variation as a result of breeding and selection. This decrease in genetic variation between Resyn lines and varieties was previously reported by studies including Resyn lines (Becker et al. 1995; Girke et al. 2011a).

In the present study, the difference in genetic distance between the Resyn lines and the *B. napus* variety groups was higher than that reported earlier (Becker et al. 1995; Girke et al. 2011a). This difference can be explained by the integration of Resyn lines that descended from wild *B. oleracea* ssp. *oleracea* and other wild *Brassica* species. Furthermore, the R_DOM_ lines had been preselected on the basis of the results of Girke et al. (2011a): Resyn lines that were genetically very distant from oilseed varieties were mainly chosen for this study.

It was unexpected that the R_DOM_ and Asian varieties were genetically less distant (0.45; Table 2) than the winter or spring varieties and R_DOM_ (0.50 and 0.53, respectively). Becker et al. (1999) and Hu et al. (2007) reported that most Asian varieties have been developed from the European gene pool, with some introgressions from Asian *B. rapa* oil and vegetable forms (Sernyk 1999; Qian et al. 2006; Hu et al. 2007). Many R_DOM_ lines are based on *B. rapa* vegetable subspecies (ssp. *chinensis*, ssp. *pekinensis*), which may explain why the genetic distance to the Asian gene pool was lower than the distance to the European gene pool (winter and spring; Table 2).

In accordance with Girke et al. (2011a), the R_DOM_ lines did not form a distinct cluster in the principal coordinate analysis or in the cluster analysis, where two clusters (Fig. 2: IVa, IVb) of the dendrogram contained almost solely R_DOM_ genotypes. The within-group variation of the R_DOM_ lines was higher (0.55) than the variation among the variety genotype groups.

Our genetic distance data indicate that the R_WILD_ lines harbor a genetic diversity not present in the breeding material or the R_DOM_ lines. R_WILD_ lines would therefore be suitable to form a heterotic gene pool for hybrid breeding that is adequately distant from the adapted *B. napus* winter oilseed rape.

### Hybrid yield and genetic distance

In the present study, heterosis could not be estimated directly because the paternal Resyn lines did not survive in the field due to their poor winter hardiness. The correlation between hybrid yield and genetic distance was negative and low, with *r* = −0.29 (*P* = 0.1). This result agrees with earlier studies: Girke et al. (2001) reported little correlation (*r* = −0.01) for hybrid yield and genetic distance in 12 hybrids of the spring variety ‘Korall’ and Resyn lines, while Girke et al. (2011b) estimated respective correlations of *r* = −0.10 and −0.23 for hybrids derived from 44 Resyn lines and two winter oilseed rape tester lines, respectively.

It is often assumed that genetic distance (based on DNA markers) and hybrid yield, as well as genetic distance and heterosis, are correlated positively. Charcosset and Essioux (1994) and Melchinger (1999) postulated that weak or no correlations would be expected if the hybrid parents originated from genetically diverse heterotic groups (inter-group hybrids).

The low or missing correlation between genetic distance and hybrid yield in inter-group crosses can be explained by differences in the linkage disequilibrium of DNA markers and trait loci in the parental heterotic groups. If the levels of linkage disequilibrium or even linkage phases differ strongly between loci in the two parental groups, the markers cannot predict heterosis or hybrid yield, and correlations between genetic distance and hybrid yield should tend to be small and non-significant. The results of this study therefore do not conflict with other studies on genetic distance and hybrid yield in oilseed rape that reported closer correlations. For example Diers et al. (1996) estimated a correlation of *r* = 0.59 between hybrid yield and genetic distance in diallelic crosses of seven spring oilseed varieties, and Riaz et al. (2001) evaluated hybrids of ten spring rapeseed lines and 12 restorer lines to detect a correlation of *r* = 0.64.

Similar observations have been reported in other species when wild or exotic genotypes were used in hybrid studies. Zeng and Meredith (2011) estimated a correlation of *r* = −0.17 between *F*_2_ performance and genetic distance (estimated on the basis of simple sequence repeat-based markers) in crosses between four elite cotton lines and 12 exotic germplasm lines. In crosses within and between groups of American and Chinese maize lines, related lines showed the highest correlations between genetic distance and grain yield (*r* = 0.47), while inter-group hybrids were not correlated (*r* = −0.09; Zheng et al. 2008).

### Use of R_WILD_ Resyn lines in *B. napus* hybrid breeding

The yield of all hybrids derived from R_WILD_ lines was lower than that of the hybrid ‘Visby’. The best R_WILD_ hybrid was ‘Tester B × MOY 4’, which yielded 89 % of the ‘Visby’ yield. Considering that R_WILD_ lines were not preselected for line per se performance, the hybrid yield can be regarded as high. However, R_WILD_ lines generally lacked agronomic performance in traits such as disease resistances, lodging, and winter hardiness.

The reduced winter hardiness of the Resyn lines hampered hybrid seed production to a great extent. The winter hardiness of the hybrids was higher than that of the Resyn parental lines. The lowest winter hardiness was observed in R_WTYPE_ Resyn lines that descended from crosses of ‘Yellow Sarson’ with wild *B*. *oleracea* ssp. *oleracea,* whereas crosses with *Brassica* wild species resulted in Resyn lines with higher winter hardiness. This observation was unexpected, since the wild *B*. *oleracea* ssp. *oleracea* represents the northernmost-distributed representatives of this species (Lannér et al. 1997; Gladis and Hammer 2003), and it was assumed that they would be better adapted to northern European winters than the *Brassica* species of Mediterranean origin.

As *B. rapa* parents, the European winter variety ‘Largo’ and the Indian spring variety ‘Yellow Sarson’ were used in the development of Resyn lines. For unknown reasons, development of the Resyn lines was much more successful with ‘Yellow Sarson’, and therefore most Resyn lines included in the field trials were of this type. It was expected that descendents from spring *B. rapa* ‘Yellow Sarson’ are less winter hardy than Resyn lines originating from the winter variety ‘Largo’, but this hypothesis could not be confirmed.

Does the *B. oleracea* origin of the Resyn lines, which were used as hybrid parents, influence hybrid yield? Wild-type and wild *B. oleracea* genotypes did not undergo domestication selection, and thus lack agronomic domestication traits like disease resistance and winter hardiness, in contrast to the co-selection for general agronomical and horticultural adaptation in *B. oleracea* vegetable breeding. It remains open for discussion whether wild *B. oleraceae* therefore has a higher seed yield than vegetable *B. oleracea*, as it was not possible to directly determine the seed yield in the *B. oleracea* genotypes or in the Resyn lines. Indirect estimation of seed yield in the hybrids revealed that the highest-yielding hybrids all originated from crosses with R_DOM_ lines (Fig. 3); the mean seed yield of these hybrids was significantly higher than the mean seed yield of hybrids from R_WTYPE_ or R_WSPEC_ lines (Table 4). However, it was unexpected, that some Resyn lines descending from wild Brassica species produced high yielding hybrids, such as ‘Tester B × MOY 4’ (Fig. 3). Bearing in mind, that the paternal line of ‘MOY 4’, *B. montana* accession ‘6835’ was collected in the year 1984 in a ruderal habitat of Gerona, Spain, and has never been preselected for any agronomic traits, the yield of the hybrid can be considered as high. Nevertheless, the resulting suggestion would not be to use *B. montana* Resyn lines as parental line in hybrid programs, but, it could be considered to use these Resyn lines to provide genetic distance to the adapted rapeseed hybrid breeding gene pools.

Genetic changes in newly formed allopolyploids may generate novel gene expression and phenotypic variation (reviewed by Pires and Gaeta 2011). We did not observe any evidence of genetic instability in our R_DOM_ lines, such as fertility problems due to the restructuring of merged genomes (Szadkowski et al. 2010). Most of the R_DOM_ lines have been propagated in the field for several generations and were preselected for agronomic performance and seed set (Girke et al. 2011b). R_WILD_ lines have been developed more recently, but we did not observe any obvious changes in fertility or phenotype. However, before introgressing Resyn lines into *B. napus* breeding programs, their genetic stability must be confirmed.

## Conclusions

When we began these investigations, we assumed that Resyn lines with wild species serving as the donor of the C genome would exhibit relatively high seed performance compared to Resyn lines from *B. oleracea* vegetable types because natural selection should favor seed production over the selection aims of artificial vegetable breeding. However, most R_WILD_ lines had very low seed performance, and most of these lines displayed very poor winter hardiness. Despite their low agronomic performance, these lines produced surprisingly high-yielding hybrids when crossed with adapted genotypes. Therefore, these R_WILD_ lines represent a suitable genetic resource for broadening the basis of oilseed rape breeding beyond what has been possible with Resyn lines from the vegetable types of *B. oleracea*.

## Electronic supplementary material

Below is the link to the electronic supplementary material.

Supplementary material 1 (DOC 393 kb)

## Abbreviations

Resyn line: Resynthesized line
