# Supplementary material for: Resynthesized lines from domesticated and wild Brassica taxa and their hybrids with B. napus L.: genetic diversity and hybrid yield
Source: Theor Appl Genet. 2013 Jan 18;126(4):1053–65. doi: 10.1007/s00122-012-2036-y (PMC3607727; doi:10.1007/s00122-012-2036-y)
Supplement: Supplementary file 1 — Supplementary material 1 (DOC 393 kb) [file 122_2012_2036_MOESM1_ESM.doc]

ESM 1: Origin of *Brassica napus* varieties and lines.

| Genotype | Accession Nr. | Type/ origin^a^ | Seed quality^b^ | Breeding company/ origin^c^ | Country | | Year of release | |  |
| --- | --- | --- | --- | --- | --- | --- | --- | --- | --- |
|  |  |  |  |  |  | |  | |  |
| Aphid Resistant Rape | CR 167 | W, V |  |  | NZ | |  | |  |
| Alesi |  | W | 00 | KWS | DE | | 2004 | |  |
| Billy |  | W | 00 | DSV | DE | | 2005 | |  |
| Campari |  | W, F | 00 | NPZ | DE | | 1996 | |  |
| Digger |  | W | 00 | KWS | DE | |  | |  |
| Emerald | CR 312 | W, F | 00 | DA | IE | | 1973 | |  |
| Express 617 |  | W | 00 | NPZ | DE | | 1993 | |  |
| Favorite |  | W | 00 | DSV | DE | | 2006 | |  |
| Gießener Höhenraps | CR 579 | W | ++ |  | DE | |  | |  |
| Jet Neuf | CR 662 | W | 0+ | Serasem | FR | | 1977 | |  |
| Ladoga |  | W | 00 | Limagrain | DE | | 2005 | |  |
| Lembkes Normal |  | W | ++ | NPZ | DE | |  | |  |
| Mansholt 54^d^ |  | W | ++ |  | DE | |  | |  |
| Mansholts Hamburger | CR 754 | W | ++ | Mansholt | NL | | 1899 | |  |
| Mosa |  | W, F | 00 | Joordens | NL | | 2001 | |  |
| Nikos |  | W, F | 00 | Euro Grass |  | |  | |  |
| Norde | CR 3104 | W | ++ | Svalöv | SE | | 1968 | |  |
| Oase |  | W | 00 | DSV | DE | | 2004 | |  |
| Samourai 11.4^d^ |  | W | 00 |  |  | |  | |  |
| Samourai | CR 176 | W | 00 | INRA/Serasem | | FE | | 1989 | |
| Sollux | CR 952 | W | ++ | Lüsewitz | DE | | 1973 | |  |
| Viking |  | W | 00 | NPZ | DE | | 2002 | |  |
| Tester A |  | W, mS | 00 | NPZ | DE | | 1999 | |  |
| Tester B |  | W, mS | 00 | Syngenta | DE | | 2009 | |  |
| Bronowski | CR 270 | S | +0 | IHAR | PL | | ~1945 | |  |
| Golden | CR 3141 | S | ++ | AG Cda | CA | | 1954 | |  |
| Heros |  | S | 00 | Raps Gbr | DE | | 2000 | |  |
| Licosmos |  | S | 00 | DSV | DE | | 1996 | |  |
| Mazowieki | CR3165 | S | ++ | IHAR | PL | | ~1945 | |  |
| Mlochowski | CR 3117 | S | ++ | IHAR | PL | | ~1945 | |  |
| Nugget | CR 3133 | S | ++ | AG Cda | CA | | 1961 | |  |
| Petranova | CR 3231 | S | ++ | Petkus | DE | | 1963 | |  |
| Regina, | CR 882 | S | ++ | Svalöv | SE | | 1942 | |  |
| Siesta |  | S | 00 | NPZ | DE | | 2003 | |  |
| Svaloefs Gulle | CR 3283 | S | ++ | Svalöv | SE | | 1969 | |  |
| Tanka | CR 1011 | S | ++ | U. of Man. | CA | | 1963 | |  |
| Tira | CR 1016 | S | ++ | Borries | DE | | 1972 | |  |
| Topas | CR 3086 | S | 00 | Svalöv | SE | | 1981 | |  |
| Westar | CR 1054 | S | 00 | AG Cda | CA | | 1982 | |  |
| Zachodni | CR 1170 | S | ++ | ZNRio | PL | | ~1945 | |  |
| Eskisehir |  | S, T |  |  | TR | |  | |  |
| Turhal |  | S, T |  |  | TR | |  | |  |
| Yenisehir |  | S, T |  |  | TR | |  | |  |
| Ganyu 3 |  | A | ++ | CAAS | CN | | 1977 | |  |
| Italy |  | A | ++ | HAU | CN | |  | |  |
| Linyou 5 |  | A | ++ | JAAS | CN | |  | |  |
| Zhenyou 11 |  | A | ++ | ZAU | CN | |  | |  |
| Xiangyou 11 |  | A | 00 | HAAS | CN | |  | |  |
| 87-50182 |  | A | ++ | WIAS | CN | |  | |  |
| Brauner Schnittkohl | BRA 175 | V (leaf) |  |  | DE | | < 1945 | |  |
| Goldgelber Zarter Butter | BRA 176 | V (leaf) |  |  | DE | | < 1945 | |  |
| Grüner Schnittkohl | BRA 177 | V (leaf) |  |  | DE | | < 1945 | |  |
| Mecklenburger Weiße | BRA 1787 | V (sw) |  |  | DE | |  | |  |
| MB6-BRS-039 |  | V (leaf) |  |  | ES | |  | |  |
| Wilhelmsb. Steckrübe |  | V (sw) |  |  | DE | |  | |  |

^a^W, winter; S, spring; T, Turkisch; A, Asian; mS, male sterile line; F, fodder type; V, vegetable; V(sw), vegetable (swede); V(leaf), vegetable (leafy cabbage).

^b^ high (+) or low (0) content of erucic acid and glucosinolates in the seeds.

^c^Origin and address, see below.

**^d^**Doubled haploid line of the respective variety .

|  | Address |
| --- | --- |
| AG Cda | Agriculture and Agri-Food Canada,  107 Science Place, Saskatoon, Saskatchewan, S7N 0X2, Canada |
| Borries | W. von Borries-Eckendorf GmbH & Co.KG  Hovedisser Straße 92, 33818 Leopoldshöhe, Germany |
| CAAS | Oil Crops Research Institute, Chinese Academy of Agricultural Science,  No2 Xudong Second Road Wuhan, Hubei Province, P.R.China 430062 |
| CGN | Centrum voor Genetische Bronnen Nederland (CGN)  Postbus 9101, 6700 HB Wageningen, Netherlands |
| DA | Department of Agriculture,  Agriculture House, Kildare St., Dublin 2, Irland |
| DSV | Deutsche Saatveredelung AG, Weissenburger Straße 5, 59557 Lippstadt Postfach 1407, 59524 Lippstadt, Germany |
| ESP | Banco de Germoplasma, Universidad Politécnica de Madrid. Escuela Técnica Superior de Ingenieros Agrónomos., Madrid, Spane |
| FUB | FU Berlin, Institut für Biologie, Angewandte Genetik,  Albrecht-Thaer-Weg 6, 14195 Berlin, Germany |
| GAU | Georg-August-Universität Göttingen, Department für Nutzpflanzenwissenschaften, Abt. Pflanzenzüchtung, Von-Siebold-Straße 8, 37075 Göttingen, Germany |
| GBR | Warwick Crop Centre, The University of Warwick,  WellesbourneWarwick, CV35 9EF, United Kingdom |
| HAAS | Hunan Agricultural University  Furong District, Changsha City, Hunan Province, P.R.China 410128 |
| HAU | Huazhong Agricultural University,  No1, Shizishan Street, Hongshan District, Wuhan, Hubei Province, P.R.China 430070 |
| IHAR | **Instytut Hodowli i Aklimatyzacji Roślin - Państwowy Instytut Badawczy** Radzików, 05-870 Błonie, Polen |
| INRA | INRA (Institute national de la recherché agronomique)  147 rue de l'université, 75338 Paris Cedex 07, France |
| IPK | Leibniz-Institut für Pflanzengenetik und Kulturpflanzenforschung (IPK)  Abt. Genbank, Corrensstraße 3, 06466 Gatersleben, Germany |
| JAAS | Jiangsu Academy of Agricutural Sciences,  50 Zhongling Street, Nanjing 210014, P.R.China |
| JLU | Justus-Liebig-Universität Gießen, IFZ, Institut für Pflanzenbau und Pflanzenzüchtung I, Heinrich-Buff-Ring 26, 35392 Gießen, Germany |
| Joordens | Joordens Zaden B.V.  Postbus 7823, 5995 ZG Kessel, Netherlands |
| KWS | KWS SAAT AG,  Grimsehlstr. 31, Postfach 1463, 37555 Einbeck, Germany |
| Limagrain | Limagrain GmbH,  Griewenkamp 2, 31234 Edemissen, Germany |
| Mansholt | DR. R.J. MANSHOLT'S VEREDELINGSBEDRIJF B.V.  Westpolder 8 PB 2 9975 WJ Vierhuizen, Germany |
| NPZ | Norddeutsche Pflanzenzucht Hans-Georg Lembke KG,  Hohenlieth, 24363 Holtsee, Germany |
| Petkus | siehe KWS |
| Raps GbR | **Raps GbR** Saatzucht Lundsgaard  Streichmühler Straße 8a, 24977 Grundhof, Germany |
| Serasem | Serasem,  60 rue Léon Beauchamp - BP 45, 59933 La Chapelle d’Armentières cedex  France |
| Svalöv  ( = SW Seed) | Lantmännen SW Seed AB,  268 81 Svalöv, Sweden |
| Syngenta | Syngenta Seeds GmbH,  Zum Knipkenbach 20, 32107 Bad Salzuflen, Germany |
| U. of Man. | Department of Plant Science, University of Manitoba,  222 Agriculture Building,66 Dafoe Road ,Winnipeg, MB, Canada - R3T 2N2 |
| VDLUFA | VDLUFA Qualitätssicherung,  Am Versuchsfeld 13, 34128 Kassel, Germany |
| WIAS | Wanxian Institute of Agricultural Sciences of Sichuan Province,  Wanxian, P.R.China 634006 |
| ZAU | ZheJiang Academy of Agriculteral Sciences,  Shiqiao Rd, Hangzhou. Zhejiang, P.R. China 310021 |
| ZNRio | see IHAR |

ESM 2: Origin of domesticated (R_DOM_) and non-domesticated Resyn (R_WSPEC_; R_WTYPE_) lines. For further information concerning R_DOM_ genotypes it is referred to Girke et al. (2011a).

| **Resyn group^a^** | **Resyn line** | | **Maternal genotype** | **Variety/ Type^b^** | **Descriptor** | **Paternal genotype** | **Variety/ Type^b^** | **Descriptor** |
| --- | --- | --- | --- | --- | --- | --- | --- | --- |
|  |  | |  |  |  |  |  |  |
| R_WSPEC_ | **BOY 1** | | *B. rapa* ssp*. trilocularis* | ‘Yellow Sarson’ | RO18 | *B. bourgaei* | WSPEC | BRA 2998 (= K 9825) |
| R_WSPEC_ | **CRL 1** | | *B. rapa* ssp*.*  *oleifera* | ‘Largo’ |  | *B. cretica*  ssp. *aegaea* | WSPEC | 6346 |
| R_WSPEC_ | **CRY 1** | | *B. rapa* ssp*. trilocularis* | ‘Yellow Sarson’ | RO18 | *B. cretica* | WSPEC | 5971 |
| R_WSPEC_ | **CRY 2** | | *B. rapa* ssp*. trilocularis* | ‘Yellow Sarson’ | RO18 | *B. cretica*  ssp. *aegaea* | WSPEC | 6021 |
| R_WSPEC_ | **HIL 1** | | *B. hilarionis* | WS | HRIGRU 12483 | *B. rapa* ssp.  *oleifera* | ‘Largo’ |  |
| R_WSPEC_ | **HIY 1** | | *B. rapa* ssp*. trilocularis* | ‘Yellow Sarson’ | RO18 | *B. hilarionis* | WSPEC | 7344 |
| R_WSPEC_ | **HIY 2** | | *B. rapa* ssp*. trilocularis* | ‘Yellow Sarson’ | A59 | *B. hilarionis* | WSPEC | HRIGRU 12483 |
| R_WSPEC_ | **INL 1** | | *B. rapa* ssp*.*  *oleifera* | ‘Largo’ |  | *B. incana* | WSPEC | 6570 |
| R_WSPEC_ | **INL 2** | | *B. rapa* ssp*.*  *oleifera* | ‘Largo’ |  | *B. incana* | WSPEC | 6572 |
| R_WSPEC_ | **INY 1** | | *B. rapa* ssp*. trilocularis* | ‘Yellow Sarson’ | RO18 | *B. incana* | WSPEC | 6563 |
| R_WSPEC_ | **INY 3** | | *B. rapa* ssp*. trilocularis* | ‘Yellow Sarson’ | RO18 | *B. incana* | WSPEC | 6569 |
| R_WSPEC_ | **INY 4** | | *B. rapa* ssp*. trilocularis* | ‘Yellow Sarson’ | RO18 | *B. incana* | WSPEC | 6570 |
| R_WSPEC_ | **INY 5** | | *B. rapa* ssp*. trilocularis* | ‘Yellow Sarson’ | RO18 | *B. incana* | WSPEC | BRA 2918  (= K 8118) |
| R_WSPEC_ | **INY 6** | | *B. rapa* ssp. *trilocularis* | ‘Yellow Sarson’ | RO18 | *B. incana* | WSPEC | CGN18470 |
| R_WSPEC_ | **ISY 1** | | *B. rapa* ssp*. trilocularis* | ‘Yellow Sarson’ | RO18 | *B. insularis* | WSPEC | 7356 |
| R_WSPEC_ | **ISY 2** | | *B. rapa* ssp*. trilocularis* | ‘Yellow Sarson’ | RO18 | *B. insularis* | WSPEC | BRA 2996 (= K 8934) |
| R_WTYPE_ | **J 104** | | *B. rapa ssp. trilocularis* | ‘Yellow Sarson’ | Syn 9 | *B. oleracea*  *ssp. oleracea* | WTYPE | BRA 259 |
| R_WTYPE_ | **J 112** | | *B. rapa ssp. trilocularis* | ‘Yellow Sarson’ | Syn 9 | *B. oleracea*  *ssp. oleracea* | WTYPE | BRA 101 |
| R_WTYPE_ | **J 134** | | *B. oleracea*  *ssp. oleracea* | WTYPE | BRA 260 | *B. rapa* | ‚NPZ 00‘ |  |
| R_WTYPE_ | **J 154** | | *B. oleracea*  *ssp. oleracea* | WTYPE | BRA 260 | *B. rapa ssp. trilocularis* | ‘Yellow Sarson’ | Syn 9 |
| R_WTYPE_ | **J 161** | | *B. rapa ssp. trilocularis* | ‘Yellow Sarson’ | Syn 9 | *B. oleracea*  *ssp. oleracea* | WTYPE | BRA 259 |
| R_WTYPE_ | **J 166** | | *B. oleracea*  *ssp. oleracea* | WTYPE | BRA 101 | *B. rapa ssp. trilocularis* | ‘Yellow Sarson’ | Syn 9 |
| R_WTYPE_ | **J 32** | | *B. rapa ssp. trilocularis* | ‘Yellow Sarson’ | Syn 9 | *B. oleracea*  ssp. *oleracea* | WTYPE | BRA 259 |
| R_WTYPE_ | **J 400** | | *B. oleracea*  *ssp. oleracea* | WTYPE | BRA 101 | *B. rapa* | ‚NPZ 00‘ |  |
| R_WTYPE_ | **J 401** | | *B. oleracea*  *ssp. oleracea* | WTYPE | BRA 260 | *B. rapa* | ‚NPZ 00‘ |  |
| R_WTYPE_ | **J 408** | | *B. oleracea*  *ssp. oleracea* | WTYPE | BRA 260 | *B. rapa* ssp. *trilocularis* | ‘Yellow Sarson’ | Syn 9 |
| R_WTYPE_ | **J 410** | | *B. rapa ssp. trilocularis* | ‘Yellow Sarson’ | Syn 9 | *B. oleracea*  ssp*. oleracea* | WTYPE | BRA 101 |
| R_WTYPE_ | **J 45** | | B. *rapa* ssp*. trilocularis* | ‘Yellow Sarson’ | Syn 9 | B. *oleracea*  ssp. *oleracea* | WTYPE | BRA 108 |
| R_WSPEC_ | **MAY 1** | | *B. rapa ssp. trilocularis* | ‘Yellow Sarson’ | RO18 | *B. macrocarpa* | WSPEC | 6584 |
| R_WSPEC_ | **MOL 1** | | *B. rapa* ssp*.*  *oleifera* | ‚Largo‘ |  | *B. montana* | WSPEC | K 7220 |
| R_WSPEC_ | **MOL 2** | | *B. rapa* ssp*.*  *oleifera* | ‚Largo‘ |  | *B. montana* | WSPEC | BRA 2946  (= K 8380) |
| R_WSPEC_ | **MOL 4** | | *B. rapa* ssp*.*  *oleifera* | ‚Largo‘ |  | *B. montana* | WSPEC | 6816 |
| R_WSPEC_ | **MOY 1** | | *B. rapa* ssp*. trilocularis* | ‘Yellow Sarson’ | RO18 | *B. montana* | WSPEC | 6801 |
| R_WSPEC_ | **MOY 2** | | *B. rapa* ssp*. trilocularis* | ‘Yellow Sarson’ | RO18 | *B. montana* | WSPEC | 6816 |
| R_WSPEC_ | **MOY 4** | | *B. rapa* ssp. *trilocularis* | ‘Yellow Sarson’ | RO18 | *B. montana* | WSPEC | 6835 |
| R_WSPEC_ | **MOY 5** | | *B. rapa* ssp*. trilocularis* | ‘Yellow Sarson’ | RO18 | *B. montana* | WSPEC | BRA 2942 (= K 7220) |
| R_WSPEC_ | **MOY 6** | | *B. rapa* ssp*. trilocularis* | ‘Yellow Sarson’ | RO18 | *B. montana* | WSPEC | CGN18472 |
| R_WSPEC_ | **MOY 7** | | *B. rapa* ssp*. trilocularis* | ‘Yellow Sarson’ | RO18 | *B. montana* | WSPEC | BRA 2993 (= K 7223) |
| R_WTYPE_ | **OLL 1** | | *B. rapa* ssp.  *oleifera* | ‚Largo‘ |  | *B. oleracea*  ssp. *oleracea* | WTYPE | BRA 258 |
| R_WTYPE_ | **OLL 1** | | *B. rapa* ssp.  *oleifera* | ‚Largo‘ |  | *B. oleracea*  ssp. *oleracea* | WTYPE | BRA 258 |
| R_WTYPE_ | **OLY 1** | | *B. rapa* ssp. *trilocularis* | ‘Yellow Sarson’ | RO18 | *B. oleracea* | WTYPE | 7660 |
| R_WTYPE_ | **OLY 2** | | *B. rapa* ssp. *trilocularis* | ‘Yellow Sarson’ | RO18 | *B. oleracea*  var*. selenisia* | TV | BRA 1668  (= K 9344) |
| R_WSPEC_ | **RUY 1** | | *B. rapa* ssp*. trilocularis* | ‘Yellow Sarson’ | RO18 | *B. rupestris*  ssp. *hispida* | WSPEC | 6580 |
| R_WSPEC_ | **TAY 1** | | *B. rapa* ssp*. trilocularis* | ‘Yellow Sarson’ | RO18 | *B. taurica* | WSPEC | BRA 2947 (= K 9238) |
| R_WSPEC_ | **VIL 1** | | *B. rapa* ssp.  *oleifera* | ‚Largo‘ |  | *B*. *villosa* | WSPEC | HRIGRU  6848 |
| R_DOM_ | **G 39^e^** | | *B. oleracea* convar. *capitata* var. *capitata* | ‘Sun Up H’ |  | *B. rapa* ssp. oleifera | Yellow seeded |  |
| R_DOM_ | **G 50^e^** | | *B. oleracea* convar. *acephala* var*. gongylodes* | ‘Erfordia’ |  | *B. rapa* ssp. oleifera | Yellow seeded |  |
| R_DOM_ | **H 44^e^** | *B. oleracea* convar. *capitata* var. *sabauda* | | ‘Eisenkopf’ |  | *B. rapa* ssp*. pekinensis* | ‘Ho46’ |  |
| R_DOM_ | **H 149^e^** | *B. oleracea* convar. *acephala* var. *medullosa* | | ‘Cavalier Rouge’ |  | *B. rapa* ssp*. chinensis* | Pak Choi |  |
| R_DOM_ | **H 165^e^** | *B. oleracea* convar. *capitata* var*. sabauda* | | Savoy |  | *B. rapa* ssp*. chinensis* | Pak Choi |  |
| R_DOM_ | **H 176^e^** | *B. oleracea* convar. *capitata* var. *Sabauda* | | ‘Praeco’ |  | *B. rapa* ssp*.*  *pekinensis* var. *laxa* | ‘Hsiao Pai Kou Pai Tsai’ |  |
| R_DOM_ | **H 355^e^** | *B. oleracea* convar. *capitata* var. *capitata* | | ‘Hybrid 0’ |  | *B. rapa* ssp*.*  *pekinensis* | ‘Spring Giant’ |  |
| R_DOM_ | **R 53^e^** | *B. oleracea* convar. *capitata* var. *sabellica* | | winter form |  | *B. rapa* ssp*.*  *pekinensis* | ‘Early  Hybrid G’ |  |
| R_DOM_ | **R 76^e^** | *B. oleracea convar. botrytis* var. *alboglabra* | | ‘966-1-4 SI |  | *B. rapa* ssp.  *oleifera* | ‘Eskisehir II’ |  |
| R_DOM_ | **R 99^e^** | *B. oleracea* convar. *capitata* var. *capitata* | | ‘Stone Head’ |  | *B. rapa* ssp*. pekinensis* | Chinese cabbage |  |
| R_DOM_ | **S 39^e^** | *B. oleracea* convar. *capitata* var. *capitata* | | S-S Cross Japan |  | *B. rapa* ssp. *chinensis* | Pak Choi |  |
| R_DOM_ | **RS 4/2^c^** | *B. rapa* | | ‘I 85-326’ |  | *B. oleracea* convar. *capitata* var*. sabellica x*  *B. oleracea* convar. *gemmifera* | ‘WS4’ |  |
| R_DOM_ | **RS 4/6^c^** | *B. rapa* ssp*. pekinensis* | | ‘Tokyo King’ |  | *B. oleracea* convar. *capitata* var*. sabellica x*  *B. oleracea* convar. *gemmifera* | ‘WS4’ |  |
| R_DOM_ | **RS 7/6^c^** | *B. rapa* ssp*. pekinensis* | | ‘Tokyo King’ |  | *B. oleracea* convar. *acephala* var. *medullosa* | ‘WS14’ |  |
| R_DOM_ | **RS 8/6^c^** | *B. rapa* ssp*. pekinensis* | | ‘Tokyo King’ |  | *B. oleracea* convar. *acephala* var. *medullosa x B. oleracea* convar. *gemmifera* | ‘WS15’ |  |
| R_DOM_ | **RS 10/7^c^** | *B. rapa* ssp. *trilocularis* | | ‘Yellow Sarson’ |  | *B. oleracea* convar. *fructicosa* | Strauch-kohl |  |
| R_DOM_ | **RS 13/6^c^** | *B. rapa* ssp*. pekinensis* | | China Kohl |  | *B. oleracea* convar. *botrytis* var*. alboglabra* | Chinese kale |  |
| R_DOM_ | **RS 239^c^** | *B. oleracea* ssp. *botrytis* var*. botrytis* | | Blumen-kohl |  | *B. rapa* ssp. *trilocularis* | ‘Yellow Sarson’ |  |
| R_DOM_ | **S13^c^** | *B. rapa* ssp. *oleifera* 4x | | oilseed rape |  | *B. oleracea* convar. *acephala* var. *medullosa* | Marrowstem kale |  |
| R_DOM_ | **B 1/3.3^d^** | *B. rapa* ssp. *oleifera* | | ‘Asko’ | CR 1469 | *B. oleracea* convar. *capitata* var. *capitata* | ‘Kashirka 202’ | 50220-1428 |
| R_DOM_ | **FS 94.3^d^** | *B. rapa* ssp*. oleifera* | | apetalous oilseed rape |  | *B. oleracea* convar. *capitata* var. *capitata* | ‘Kashirka 202’ | 50220-1428 |
| R_DOM_ | **K 160.1.1^d^** | *B. rapa* ssp*. pekinensis* | | Chinese cabbage | 13444 | *B. oleracea* convar. *acephala* | Fodder kale | HRIGRU 8207 |
| R_DOM_ | **K 199.16.2^d^** | *B. rapa* ssp*. chinensis* | | Pak Choi | 56515 | *B. oleracea* convar. *botrytis* var. *botrytis* | *‘Maxima’* | BRA 1398 |
| R_DOM_ | **S 108.1.1^d^** | *B. rapa* ssp*. oleifera* | | high oleic oilseed rape | 98-39157 | *B. oleracea* convar. *botrytis* var. *alboglabra* | ‘Kailan’ | CGN 14044 |
| R_DOM_ | **S 228.8.1^d^** | *B. rapa* ssp*. chinensis* | | Pak Choi | 56515 | *B. oleracea* convar. *acephala* | Fodder kale | HRIGRU 8207 |
| R_DOM_ | **S 237.20.1^d^** | *B. rapa* ssp*. pekinensis* | | Chinese cabbage | 13444 | *B. oleracea* convar. *botrytis* var*. botrytis* | *‘Maxima’* | BRA 1398 |
| R_DOM_ | **S 45.2.2^d^** | *B. rapa* ssp*. oleifera* | | high oleic oilseed rape | 98-39188-9 | B. oleracea convar. botrytis var. italica | Broccoli | HRIGRU 7518 |

^a^ R_DOM_, Resyn line derived from domesticated *B. oleracea*; R_WTYPE_, derived from wild-type *B. oleracea*; R_WSPEC_, Resyn line derived from *B. oleracea* wild species

^b^ WTYPE, *B. oleracea* Wild-type; WSPEC, *B. oleracea* wild species; TV, traditional variety.

^c^ received from Freie Universität Berlin, Germany

^d^ received from Justus-Liebig-Universität Gießen, Germany

^e^ originated from Georg August Universität Göttingen, Germany

^f^ developed by José Die, Birgit Olberg, Tobias Jesske, and Friedrich Kopisch-Obuch at Georg August Universität Göttingen, Germany.

ESM 3: Mean yield and agronomic characters of hybrids with ‘Tester A’ and ‘Tester B’ and three checks, (a) evaluated at four locations in the season 2009-2010, and (b) evaluated at nine locations in the season 2009-2010.

|  | **(a)** |  |  |  |  |  |  |  |  |  | **(b)** |  |  |  |  |  | |  |  |  | |  |  |
| --- | --- | --- | --- | --- | --- | --- | --- | --- | --- | --- | --- | --- | --- | --- | --- | --- | --- | --- | --- | --- | --- | --- | --- |
| Genotype | Yield  (dt ha^-1^) | Oil (%) | Erucic acid (%) | Protein (%) | GSL^a^ | TSW^b^  (g) | Plant height  (cm) | BF^c^ | WH^d^ | Lodging  (1-9) | Yield  (dt ha^-1^) | Oil (%) | Erucic acid (%) | Protein (%) | GSL^a^ | TSW^b^  (g) | | Plant height  (cm) | BF^c^ | WH^d^ | | Lodging  (1-9) |  |
|  |  |  |  |  |  |  |  |  |  |  |  |  |  |  |  |  | |  |  |  | |  |  |
| Tester A × B 1/3.3 | 38.6 | 44.1 | 11.7 | 18.7 | 49.7 | 4.2 | 163.8 | 116.3 | 2.5 | 2.0 | 40.5 | 43.8 | 11.2 | 19.2 | 50.8 | 4.4 | 175.0 | | 117.0 | | 3.3 | 2.0 | |
| Tester A × BOY 1 | 31.6 | 42.4 | 16.2 | 19.8 | 55.4 | 4.7 | 152.5 | 117.0 | 3.5 | 3.0 | - | - | - | - | - | - | - | | - | | - | - | |
| Tester A× FS 94.3 | 41.6 | 43.2 | 3.1 | 18.4 | 34.3 | 4.3 | 166.3 | 117.3 | 2.5 | 2.0 | 41.7 | 42.8 | 3.1 | 18.9 | 34.2 | 4.4 | 172.5 | | 118.3 | | 3.1 | 2.6 | |
| Tester A × G 39 | 37.8 | 42.2 | 11.7 | 20.0 | 51.7 | 4.4 | 161.3 | 119.3 | 2.5 | 3.7 | 37.9 | 42.4 | 10.4 | 20.0 | 51.7 | 4.4 | 166.3 | | 120.0 | | 3.3 | 3.7 | |
| Tester A × G 50 | 41.9 | 43.8 | 1.7 | 18.6 | 38.3 | 4.3 | 158.8 | 117.0 | 2.0 | 2.3 | 41.7 | 43.8 | 1.0 | 18.5 | 37.5 | 4.3 | 163.8 | | 117.8 | | 2.9 | 1.9 | |
| Tester A × H 149 | 45.0 | 45.5 | 24.5 | 17.8 | 44.6 | 4.3 | 155.0 | 113.0 | 2.0 | 3.0 | 46.6 | 45.1 | 22.8 | 18.3 | 44.8 | 4.3 | 165.6 | | 114.3 | | 2.6 | 3.1 | |
| Tester A × H 165 | 37.7 | 44.0 | 13.2 | 19.2 | 37.8 | 4.1 | 151.3 | 117.7 | 3.3 | 3.0 | 39.2 | 44.1 | 13.8 | 19.3 | 38.5 | 4.1 | 156.3 | | 118.5 | | 3.6 | 2.3 | |
| Tester A × H 176 | 47.1 | 45.6 | 16.1 | 18.1 | 27.7 | 4.5 | 162.5 | 117.0 | 2.5 | 2.0 | 49.5 | 45.4 | 15.4 | 18.2 | 26.9 | 4.6 | 165.6 | | 118.2 | | 3.0 | 1.9 | |
| Tester A × H 44 | 41.7 | 45.1 | 24.9 | 19.6 | 32.9 | 4.6 | 152.5 | 111.0 | 2.3 | 1.7 | 44.2 | 44.5 | 22.8 | 19.7 | 33.5 | 4.7 | 157.5 | | 113.0 | | 2.9 | 1.9 | |
| Tester A × INL 2 | 34.4 | 41.5 | 12.8 | 20.4 | 44.8 | 4.9 | 155.0 | 115.7 | 2.3 | 2.3 | - | - | - | - | - | - | - | | - | | - | - | |
| Tester A × J 134 | 39.5 | 43.1 | 11.1 | 19.0 | 32.8 | 4.6 | 147.5 | 112.0 | 2.0 | 2.7 | - | - | - | - | - | - | - | | - | | - | - | |
| Tester A × J 154 | 33.8 | 42.3 | 17.7 | 19.7 | 53.7 | 5.2 | 148.8 | 108.3 | 3.0 | 2.3 | 35.8 | 42.3 | 15.0 | 19.8 | 49.8 | 5.1 | 154.4 | | 110.3 | | 3.9 | 2.3 | |
| Tester A × J 161 | 34.4 | 44.2 | 19.3 | 18.2 | 38.2 | 4.0 | 137.5 | 115.3 | 3.5 | 4.3 | 33.2 | 43.6 | 17.0 | 18.8 | 36.9 | 4.1 | 145.6 | | 116.7 | | 4.5 | 3.6 | |
| Tester A × J 166 | 30.4 | 41.0 | 19.5 | 19.9 | 50.8 | 4.9 | 145.0 | 114.0 | 3.5 | 4.7 | - | - | - | - | - | - | - | | - | | - | - | |
| Tester A × J 32 | 38.3 | 43.1 | 10.7 | 18.8 | 35.5 | 4.6 | 160.0 | 114.0 | 2.5 | 2.3 | - | - | - | - | - | - | - | | - | | - | - | |
| Tester A × J 401 | 41.5 | 43.4 | 14.2 | 18.3 | 32.1 | 4.9 | 157.5 | 114.0 | 2.5 | 2.3 | 41.9 | 42.7 | 12.4 | 19.1 | 31.9 | 4.9 | 162.5 | | 115.0 | | 3.5 | 2.4 | |
| Tester A × J 45 | 37.0 | 42.9 | 17.5 | 18.9 | 40.2 | 4.4 | 147.5 | 112.3 | 3.5 | 3.0 | - | - | - | - | - | - | - | | - | | - | - | |
| Tester A × K 199.16.2 | 36.2 | 43.7 | 19.9 | 19.4 | 43.4 | 4.1 | 157.5 | 117.3 | 2.5 | 2.3 | - | - | - | - | - | - | - | | - | | - | - | |
| Tester A × MOL 1 | 35.5 | 42.0 | 13.3 | 19.8 | 53.3 | 4.7 | 162.5 | 117.3 | 2.8 | 2.3 | 39.4 | 42.0 | 11.3 | 19.9 | 53.8 | 4.6 | 163.1 | | 118.5 | | 3.3 | 2.4 | |
| Tester A × MOL 2 | 38.8 | 40.4 | 10.7 | 21.0 | 55.9 | 5.1 | 168.8 | 120.3 | 3.5 | 2.0 | 44.9 | 40.7 | 10.4 | 21.0 | 55.9 | 5.0 | 171.3 | | 120.2 | | 3.0 | 1.9 | |
| Tester A × MOY 5 | 18.4 | 41.3 | 16.4 | 20.7 | 55.8 | 4.5 | 161.3 | 114.3 | 3.0 | 2.7 | - | - | - | - | - | - | - | | - | | - | - | |
| Tester A × R 53 | 39.1 | 45.1 | 21.6 | 19.3 | 35.7 | 4.5 | 158.8 | 115.7 | 2.3 | 1.7 | 43.2 | 45.3 | 21.3 | 19.2 | 34.7 | 4.6 | 161.3 | | 116.7 | | 2.1 | 2.0 | |
| Tester A × R 99 | 38.7 | 43.9 | 7.3 | 18.7 | 30.1 | 4.4 | 158.8 | 115.0 | 2.3 | 1.7 | 42.4 | 43.7 | 7.1 | 19.1 | 30.6 | 4.3 | 166.3 | | 115.7 | | 2.9 | 1.7 | |
| Tester A × RS13/6 | 37.6 | 44.6 | 19.9 | 18.8 | 42.2 | 4.7 | 151.3 | 106.7 | 2.8 | 2.7 | - | - | - | - | - | - | - | | - | | - | - | |
| Tester A × RS 4/2 | 38.2 | 43.9 | 19.7 | 19.1 | 39.7 | 4.2 | 163.8 | 114.3 | 3.0 | 3.7 | - | - | - | - | - | - | - | | - | | - | - | |
| Tester A × S 108.1.1 | 45.3 | 43.8 | 5.3 | 18.0 | 35.5 | 4.5 | 156.3 | 108.7 | 2.0 | 1.7 | - | - | - | - | - | - | - | | - | | - | - | |
| Tester A × S 13 | 45.0 | 46.2 | 25.1 | 18.2 | 60.2 | 4.1 | 152.5 | 115.3 | 2.3 | 2.7 | 46.7 | 46.2 | 23.9 | 18.2 | 58.0 | 4.2 | 156.9 | | 116.5 | | 2.4 | 2.6 | |
| Tester A × S 228.8.1 | 37.8 | 44.1 | 15.5 | 18.9 | 39.4 | 4.3 | 156.3 | 115.0 | 2.0 | 2.3 | - | - | - | - | - | - | - | | - | | - | - | |
| Tester A × S 45.2.2 | 40.7 | 43.8 | 16.1 | 19.5 | 41.3 | 4.1 | 155.0 | 111.7 | 3.8 | 2.7 | 38.5 | 43.3 | 14.7 | 19.9 | 40.6 | 4.2 | 155.0 | | 113.3 | | 3.9 | 2.3 | |
| Tester A × VIL 1 | 40.1 | 42.7 | 12.4 | 19.2 | 40.3 | 4.9 | 162.5 | 114.0 | 2.5 | 1.3 | 39.4 | 42.4 | 11.5 | 19.6 | 38.8 | 4.9 | 168.6 | | 115.8 | | 3.1 | 1.9 | |
| Tester B × B 1/3.3 | 39.6 | 42.9 | 11.2 | 18.9 | 49.4 | 4.6 | 165.0 | 116.7 | 2.3 | 2.3 | 39.6 | 42.5 | 10.8 | 19.4 | 50.2 | 4.6 | 173.8 | | 118.0 | | 3.0 | 2.1 | |
| Tester B × BOY 1 | 38.6 | 41.6 | 16.1 | 19.2 | 52.7 | 4.8 | 160.0 | 117.7 | 3.3 | 2.7 | - | - | - | - | - | - | - | | - | | - | - | |
| Tester B × G 39 | 38.7 | 41.9 | 7.2 | 19.0 | 42.6 | 4.6 | 166.3 | 117.3 | 2.3 | 2.3 | 40.4 | 42.0 | 6.1 | 19.0 | 38.9 | 4.7 | 173.8 | | 119.0 | | 2.6 | 2.0 | |
| Tester B × G 50 | 35.7 | 43.4 | 1.8 | 17.8 | 35.5 | 4.3 | 155.0 | 117.0 | 1.8 | 2.0 | 42.1 | 43.3 | 0.9 | 18.0 | 35.5 | 4.4 | 164.4 | | 118.0 | | 2.3 | 2.1 | |
| Tester B × H 149 | 42.6 | 43.7 | 23.3 | 18.4 | 46.0 | 4.6 | 156.3 | 111.3 | 2.3 | 2.7 | 46.2 | 43.9 | 21.1 | 18.4 | 44.0 | 4.6 | 160.0 | | 113.2 | | 2.1 | 3.6 | |
| Tester B × H 165 | 39.9 | 43.3 | 12.7 | 19.0 | 34.9 | 4.1 | 148.8 | 118.0 | 2.5 | 3.0 | 38.0 | 43.1 | 12.9 | 19.3 | 34.5 | 4.1 | 156.3 | | 119.5 | | 3.1 | 2.4 | |
| Tester B × H 176 | 42.7 | 43.9 | 12.5 | 18.0 | 27.7 | 4.8 | 160.0 | 116.3 | 2.8 | 2.3 | 47.1 | 43.8 | 12.4 | 18.2 | 27.3 | 4.8 | 164.4 | | 117.7 | | 2.8 | 2.1 | |
| Tester B × H 355 | 36.3 | 42.5 | 16.6 | 19.5 | 46.7 | 4.9 | 152.5 | 115.7 | 3.0 | 2.7 | - | - | - | - | - | - | - | | - | | - | - | |
| Tester B × H 44 | 42.6 | 45.0 | 21.3 | 18.5 | 29.8 | 4.7 | 155.0 | 112.0 | 2.8 | 2.3 | 44.3 | 44.5 | 20.2 | 19.0 | 30.1 | 4.8 | 163.1 | | 113.8 | | 2.9 | 2.0 | |
| Tester B × INL 1 | 42.7 | 41.5 | 8.7 | 19.0 | 31.4 | 5.2 | 165.0 | 117.0 | 2.8 | 2.3 | - | - | - | - | - | - | - | | - | | - | - | |
| Tester B × INL 2 | 38.9 | 41.7 | 11.3 | 19.4 | 42.8 | 4.8 | 155.0 | 114.7 | 1.8 | 2.3 | 40.7 | 41.6 | 8.6 | 19.5 | 41.8 | 4.9 | 163.1 | | 115.8 | | 2.3 | 2.1 | |
| Tester B × J 112 | 38.6 | 42.9 | 15.3 | 18.4 | 36.3 | 4.6 | 147.5 | 113.7 | 3.0 | 3.3 | - | - | - | - | - | - | - | | - | | - | - | |
| Tester B × J 134 | 38.4 | 42.5 | 11.0 | 18.3 | 30.2 | 4.7 | 146.3 | 114.0 | 2.8 | 3.3 | 41.5 | 42.6 | 10.1 | 18.6 | 30.8 | 4.8 | 154.4 | | 115.0 | | 3.1 | 2.9 | |
| Tester B × J 154 | 39.5 | 42.9 | 8.1 | 18.0 | 36.8 | 4.8 | 147.5 | 108.3 | 2.5 | 2.3 | - | - | - | - | - | - | - | | - | | - | - | |
| Tester B × J 161 | 37.2 | 42.9 | 11.1 | 17.7 | 25.5 | 4.4 | 147.5 | 114.7 | 2.0 | 3.0 | 40.7 | 42.6 | 10.6 | 18.3 | 28.5 | 4.3 | 152.5 | | 116.2 | | 2.8 | 2.9 | |
| Tester B × J 166 | 36.1 | 41.7 | 15.8 | 18.6 | 43.5 | 4.7 | 150.0 | 113.3 | 4.5 | 4.7 | - | - | - | - | - | - | - | | - | | - | - | |
| Tester B × J 401 | 37.7 | 42.2 | 11.0 | 18.5 | 34.0 | 5.3 | 157.5 | 115.0 | 2.3 | 3.0 | 41.5 | 41.7 | 10.0 | 18.9 | 32.5 | 5.3 | 165.0 | | 115.7 | | 2.6 | 2.6 | |
| Tester B × J 408 | 36.6 | 42.2 | 22.0 | 18.6 | 42.9 | 4.4 | 150.0 | 112.7 | 3.8 | 5.0 | 39.7 | 42.2 | 17.8 | 18.8 | 42.4 | 4.3 | 153.8 | | 113.5 | | 4.4 | 2.7 | |
| Tester B × J 410 | 42.9 | 42.3 | 14.5 | 18.5 | 36.1 | 4.4 | 150.0 | 112.3 | 3.0 | 3.3 | - | - | - | - | - | - | - | | - | | - | - | |
| Tester B × J 45 | 38.5 | 43.3 | 10.4 | 17.6 | 30.2 | 4.4 | 152.5 | 116.3 | 3.0 | 3.0 | - | - | - | - | - | - | - | | - | | - | - | |
| Tester B × MOY 4 | 43.7 | 42.5 | 6.8 | 18.7 | 31.3 | 4.5 | 157.5 | 116.0 | 2.5 | 2.0 | - | - | - | - | - | - | - | | - | | - | - | |
| Tester B × OLL 1 | 36.9 | 41.5 | 8.2 | 19.6 | 30.1 | 5.4 | 152.5 | 114.7 | 3.0 | 3.7 | - | - | - | - | - | - | - | | - | | - | - | |
| Tester B × R 53 | 41.7 | 44.1 | 20.5 | 19.0 | 34.0 | 4.7 | 155.0 | 116.7 | 1.8 | 2.3 | 44.1 | 43.9 | 19.4 | 19.2 | 34.1 | 4.8 | 163.1 | | 117.2 | | 2.5 | 2.0 | |
| Tester B × R 99 | 44.1 | 43.2 | 5.2 | 18.2 | 27.3 | 4.4 | 156.3 | 115.7 | 2.3 | 2.0 | 46.6 | 43.1 | 4.7 | 18.5 | 28.8 | 4.4 | 161.9 | | 116.8 | | 2.3 | 2.4 | |
| Tester B × RS 10/7 | 43.7 | 43.7 | 11.4 | 18.4 | 40.9 | 4.8 | 162.5 | 116.0 | 2.5 | 2.3 | - | - | - | - | - | - | - | | - | | - | - | |
| Tester B × RS 13/6 | 38.9 | 44.0 | 15.6 | 17.9 | 36.8 | 4.5 | 160.0 | 107.7 | 1.8 | 2.0 | - | - | - | - | - | - | - | | - | | - | - | |
| Tester B × RS 4/2 | 43.6 | 43.5 | 11.5 | 18.4 | 30.8 | 4.3 | 167.5 | 115.7 | 3.0 | 2.7 | 45.3 | 43.0 | 10.3 | 18.9 | 30.9 | 4.4 | 171.3 | | 117.2 | | 3.0 | 2.4 | |
| Tester B × RS 7/6 | 41.5 | 43.3 | 20.5 | 19.4 | 35.4 | 4.9 | 156.3 | 111.3 | 2.0 | 2.3 | 42.2 | 43.4 | 19.6 | 19.6 | 37.5 | 4.9 | 161.3 | | 112.7 | | 2.5 | 2.4 | |
| Tester B × RS 8/6 | 42.0 | 43.2 | 11.7 | 19.1 | 33.0 | 5.0 | 158.8 | 115.7 | 2.5 | 2.3 | 44.0 | 43.0 | 8.7 | 18.9 | 31.7 | 5.0 | 163.1 | | 117.2 | | 2.5 | 2.1 | |
| Tester B × RUY 1 | 38.5 | 43.1 | 5.4 | 18.2 | 28.7 | 4.6 | 158.8 | 117.3 | 2.8 | 3.0 | - | - | - | - | - | - | - | | - | | - | - | |
| Tester B × S 108.1.1 | 42.3 | 41.9 | 7.6 | 19.0 | 35.5 | 4.8 | 153.8 | 108.3 | 2.8 | 3.3 | 44.3 | 42.1 | 7.9 | 19.1 | 35.1 | 4.8 | 156.9 | | 110.2 | | 2.9 | 3.3 | |
| Tester B × S 13 | 43.4 | 44.5 | 20.2 | 17.5 | 48.3 | 4.5 | 150.0 | 116.0 | 2.0 | 2.7 | 47.9 | 44.5 | 22.4 | 17.8 | 52.4 | 4.5 | 158.1 | | 117.2 | | 2.4 | 3.3 | |
| Tester B × S 39 | 44.5 | 42.0 | 0.9 | 18.0 | 18.7 | 4.4 | 147.5 | 116.7 | 2.3 | 2.3 | 44.8 | 42.1 | 0.4 | 18.0 | 18.1 | 4.5 | 154.4 | | 117.8 | | 2.5 | 2.6 | |
| Tester B × S 45.2.2 | 34.2 | 42.3 | 8.4 | 19.2 | 32.6 | 4.2 | 153.8 | 113.3 | 2.3 | 2.7 | 37.6 | 42.2 | 7.2 | 19.4 | 32.0 | 4.4 | 161.9 | | 115.2 | | 2.5 | 2.3 | |
| Fertile line tester A | 35.8 | 45.4 | 1.3 | 17.9 | 16.9 | 4.4 | 142.5 | 117.3 | 1.8 | 1.3 | 37.8 | 45.2 | 1.4 | 18.3 | 17.7 | 4.5 | 148.8 | | 118.2 | | 2.0 | 1.7 | |
| Fertile line tester B | 33.7 | 42.1 | 2.3 | 18.5 | 21.6 | 4.7 | 150.0 | 121.4 | 2.3 | 3.0 | 38.7 | 42.0 | 1.7 | 18.8 | 21.8 | 4.7 | 156.9 | | 124.7 | | 2.6 | 2.9 | |
| Visby | 49.2 | 43.6 | 0.0 | 17.0 | 15.0 | 4.8 | 157.5 | 117.0 | 2.0 | 1.7 | 51.2 | 43.8 | 0.0 | 17.0 | 15.3 | 4.8 | 161.3 | | 117.8 | | 1.9 | 1.9 | |
| LSD (p = 0.05) | 6.3 | 1.2 | 4.9 | 1.0 | 6.5 | 0.3 | 10.8 | 3.6 | 1.5 | 1.6 | 4.9 | 0.7 | 2.4 | 0.6 | 3.1 | 0.2 | 6.9 | | 1.8 | | 1.0 | 0.9 | |
| Minimum | 18.4 | 40.4 | 0.0 | 17.0 | 15.0 | 4.0 | 137.5 | 106.7 | 1.8 | 1.3 | 33.2 | 40.7 | 0.0 | 17.0 | 15.3 | 4.1 | 145.6 | | 110.2 | | 1.9 | 1.7 | |
| Maximum | 49.2 | 46.2 | 25.1 | 21.0 | 60.2 | 5.4 | 168.8 | 121.4 | 4.5 | 5.0 | 51.2 | 46.2 | 23.9 | 21.0 | 58.0 | 5.3 | 175.0 | | 124.7 | | 4.5 | 3.7 | |
| Mean of test hybrids | 39.2 | 43.1 | 13.5 | 18.8 | 38.9 | 4.6 | 155.7 | 114.6 | 2.6 | 2.7 | 42.2 | 43.2 | 12.5 | 19.0 | 38.1 | 4.6 | 162.1 | | 116.2 | | 2.9 | 2.4 | |
| Mean of checks | 39.6 | 43.7 | 1.2 | 17.8 | 17.8 | 4.6 | 150.0 | 118.6 | 2.0 | 2.0 | 42.6 | 43.7 | 1.1 | 18.0 | 18.3 | 4.6 | 155.6 | | 120.2 | | 2.2 | 2.1 | |

^a^ GSL, Glucosinolate content in µmol/g degreased seed meal

^b^ TSW, Thousend-seed weight

^c^ BF, Beginning of flowering in number of days in the year 2010

^d^ WH, for the definition of winter hardiness see Material and Methods
